# Supplementary material for: Systemic Pulmonary Events Associated with Myelodysplastic Syndromes: A Retrospective Multicentre Study
Source: J Clin Med. 2021 Mar 10;10(6):1162. doi: 10.3390/jcm10061162 (PMC7999053; doi:10.3390/jcm10061162)
Supplement: Supplementary file 1 [file jcm-10-01162-s001.pdf]

Journal of
Clinical Medicine

MDPI

Online supplementary material S1:

Procedure

The following methodology was applied in order to obtain the included patients:

A. Diagnostic inclusion criteria:

- Patients with at least 1 "*Résumé d'Unité Médicale*[1] " with the following codes in the main, related or associated diagnostic position (according to the International Statistical Classification of Diseases and Related Health Problems 10th Revision ICD-10):
    - Myelodysplastic/myeloproliferative neoplasms: C94.6; C93.1; C92.2; C93.3;
    - Myelodysplastic syndromes: D46.0-D46.9; D75.8;

AND

- Among these hospitalizations, those for which at least 1 "Résumé d'Unité Médicale[§]" also presented a diagnosis of "pulmonary pathologies" among the following ICD-10 codes:
    - Infectious: A15.0-A16.9; A19.0-A19.9; J09-J18; J20-J22; J40-J44;
    - Toxic: J70.2-J70.4;
    - Interstitial and others: J82; J84.0-J91; J92.9; J94.0-J94.9; J98.8-J98.9;
    - Tumours: C34.0-C34.9; C38.4; C38.8; D02.2; D14.3; D19.0;
    - Pulmonary symptoms: R04.2; R04.8; R04.9; R91;
    - Pulmonary hypertension: I27.0; I27.9.

B. Inclusion centres:

- Lille University hospital via its internal medicine and clinical immunology departments;
- Hospital of Armentières via all of its departments;

[1] In the French national health care system, each hospitalization is coded into a combination of medical procedures and diagnoses by the hospital concerned, in order to be remunerated accordingly.

- The two hospitals (Hôpital Saint-Philibert de Lomme and Hôpital Saint-Vincent de Lille) of the Catholic University of Lille via their oncology, haematology, pneumonology, geriatric and polyvalent medicine departments.

C. Inclusion period:

- From 01/01/2007 to 31/12/2017;
- Thereafter, follow-up data until August 2019 for patients still alive and not lost to follow-up.

D. Exclusion criteria:

Due to the retrospective nature of the study, coding errors were sometimes found. Thus, after consulting the medical records, patients were excluded if:

- MDS had worsened to acute myeloid leukaemia when the pulmonary event occurred.
- The diagnosis of MDS was not confirmed on reading the medical file, or if it could not be classified in accordance with the revised World Health Organization classification of myeloid neoplasms 2016.
- There was no characterizable pulmonary event on reading the medical file (for example, cough or dyspnoea without established diagnosis or without having been sufficiently explored to our knowledge).
- The pulmonary event was cardiogenic acute oedema of the lungs.
- The information available was insufficient.

Table S1: Details of pathogens identified during infectious pneumonias in myelodysplastic patients

**Bacteria**[1]			
Streptococcus pneumoniae	2	*Hafnia alvei*	1
Streptococcus A beta-haemolytic	1	*Escherichia coli*	2
Streptococcus alpha-haemolytic	1	*Enterobacter aerogenes*	1
Staphylococcus aureus	3	*Enterococcus faecalis*	1
Staphylococcus epidermidis	1	*Citrobacter*	1
Mycoplasma pneumoniae	2	*S.* alpha-haemolytic / *S. hominis*	1
Klebsiella pneumoniae	3	*S. aureus* / *Pseudomonas aeruginosa*	1
Klebsiella oxytoca	1	*S. aureus* / *Klebsiella oxytoca*	1
**Fungi**			
Pneumocystis jiiroveci	3	*Aspergillus fumigatus*	1
Candida glabrata	1	*Aspergillus sp.*	1
**Mycobacteria**		**Virus**	
Mycobacterium kansasii	1	Epstein-Barr virus	1

[1]: For three pneumonias, documented antibiotic therapy was reported. This means that a bacterium had been identified, but the corresponding information was not found when the medical file was checked retrospectively.

Table S2: Details of treatments received for myelodysplastic syndromes

patients	follow-up (months)	MDS	Treatment	introduction (month after T0)	discontinuation (month after T0)
55	184	T0			
54	138	T0			
53	127	T0			
52	125	T0	imatinib (50mg/d)		
51	102	T0			
50	91	T0			
49	91	T0			
48	84	T0	Danatrol 400mg/d		
47	80	T0	Danatrol 600mg/d		
46	71	T0	lenaledomide (10mg/d)	36	48
45	65	T0	azacitidine 7d/28	27	-
44	60	T0			
43	56	T0			
42	55	T0			
41	54	T0	azacitidine 30mg/m2, 5d/28		
40	44	T0			
39	42	T0	azacitidine 7d/28	0	42
38	38	T0	azacitidine 7d/28	12	38
37	35	T0	azacitidine 7d/28	29	35
36	31	T0			
35	31	T0	ALS + ciclosporine		
34	29	T0	disulone		
33	29	T0	ASCT, then Fludarabine+methotrexate +azacitidine, then azacitidine+dépakine+vezanoide	3	29
32	28	T0			
31	27	T0	azacitidine 75mg/m²		
30	27	T0			
29	27	T0			
28	26	T0			
27	24	T0	Hydrea 500mg/j	19	
26	23	T0	azacitidine 75m/m² 7d/28		
25	23	T0	azacitidine 7d/28	15	23
24	23	T0	azacitidine 7d/28		
23	18	T0			
22	17	T0	azacitidine 7d/28	8	17
21	16	T0			

20	16	T0			
19	15	T0			
18	13	T0			
17	12	T0	azacitidine 7d/28		
16	12	T0	azacitidine 7d/28	2	12
15	12	T0	azacitidine 7d/28	0	12
14	8	T0			
13	8	T0			
12	7	T0	Hydrea (1d/2)	-36	-34
11	6	T0			
10	5	T0			
9	5	T0			
8	4	T0	azacitidine 75mg/m², 7d/28		
7	4	T0			
6	3	T0			
5	3	T0			
4	3	T0			
3	2	T0			
2	2	T0			
1	1	T0			

Figures S1:

Univariate analyses of the predictive factors of post-MDS survival.

Factors	p value
Age at MDS diagnosis	0.08
Gender	**0.05**
Smoking	0.84
Inclusion centre	0.11
Primary versus secondary MDS	0.29
**IPSS**	**< 0.01**
**IPSS-R**	**< 0.01**
Charlson index[1]	0.85
**CIRS-G[2]**	**0.02**
**ACE-27**	**< 0.01**
ADs	0.37

MDS: myelodysplastic syndrome; IPSS(-R): (Revised) International Prognostic Scoring System; CIRS-G: Cumulative Illness Rating Scale for Geriatrics; ACE-27: Adult Comorbidity Evaluation-27; ADs: associated autoimmune or auto-inflammatory diseases.

[1]: the updated age-adjusted Charlson index, according to Bannay et al. 2016 Med. Care[12] <5 versus ≥5; [2]: ≤6 versus >6.

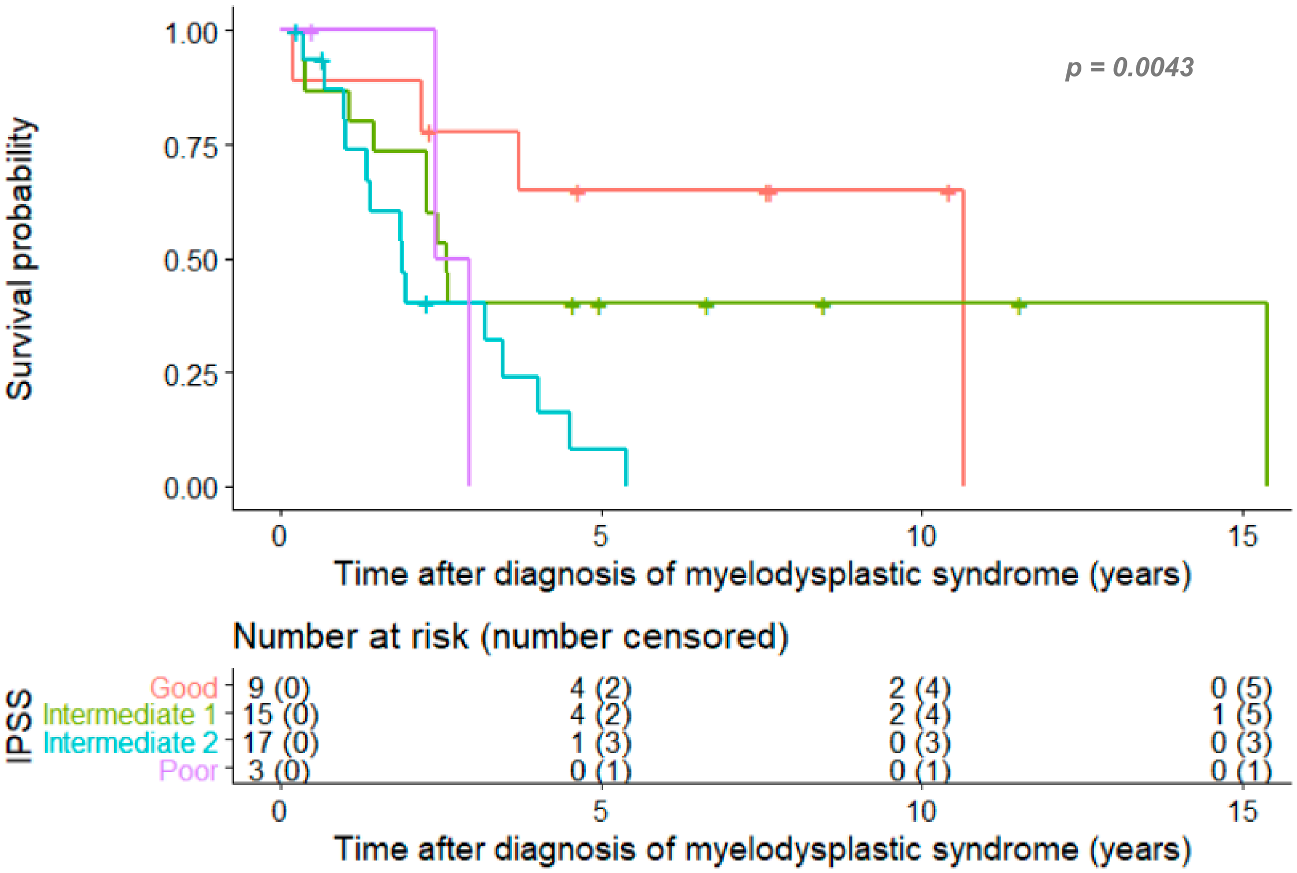

Kaplan-Meier curves for the International Prognostic Scoring System

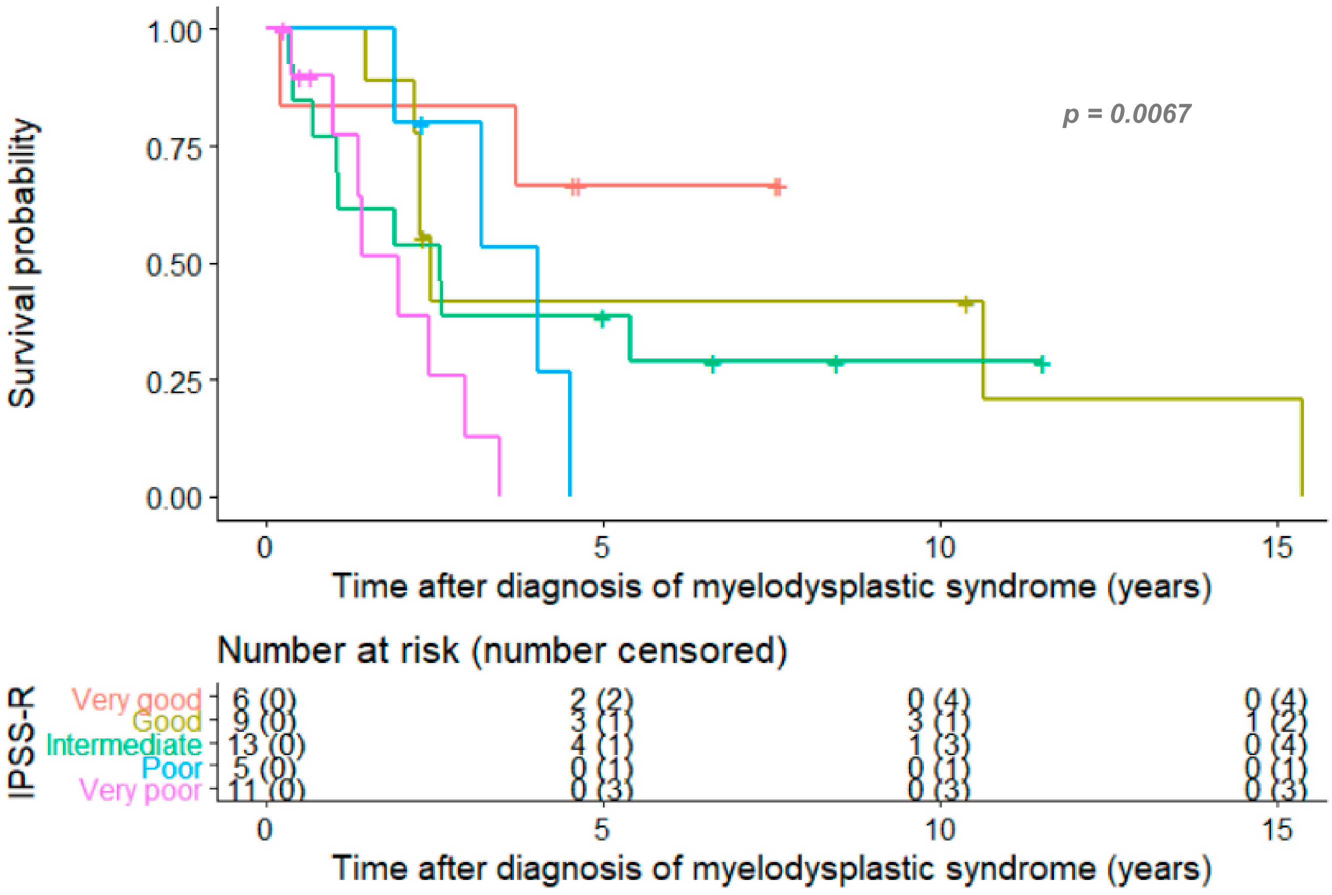

Kaplan-Meier curves for the Revised International Prognostic Scoring System
